# Barriers to breast and cervical cancer screening uptake among Black, Asian, and Minority Ethnic women in the United Kingdom: evidence from a mixed-methods systematic review

**DOI:** 10.1186/s12913-023-09410-x

**Published:** 2023-04-22

**Authors:** Obasanjo Afolabi Bolarinwa, Nicole Holt

**Affiliations:** 1 Department of Public Health & Well-Being, Faculty of Health & Social Care, University of Chester, Chester, UK; 2 Institute for Advanced Studies in the Humanities, University of Edinburgh, Edinburgh, UK; 3 Discipline of Public Health Medicine, School of Nursing and Public Health, University of KwaZulu-Natal, Durban, South Africa; 4 Department of Allied and Public Health, Faculty of Medicine, Health and Social Care, Canterbury Christ Church University, Canterbury, UK

**Keywords:** Breast cancer, Cervical cancer, Screening uptake, Black Asian and Minority Ethnic women, United Kingdom

## Abstract

**Background:**

Cancer is currently the leading cause of mortality globally, with new cancer cases estimated at 19.3 million and almost 10 million deaths in 2020. Specifically, breast and cervical cancer incidence and mortality prevalence among women of the minority group or marginalised populations in Europe have continued to be a public health concern due to the low uptake of cancer screening. Thus, this study utilised a mixed-method systematic review to identify barriers to breast and cervical screening uptake among Black, Asian, and Minority Ethnic women in the United Kingdom.

**Methods:**

Databases including PubMed, CINAHL, British Nursing Index, Web of Science, EMBASE, and Scopus databases, were systematically searched for studies on barriers to breast and cervical screening uptake among Black, Asian, and Minority Ethnic women in the United Kingdom published in English between January 2010 to July 2022. This mixed-method systematic review followed the Preferred Reporting Items for Systematic Reviews and Meta-Analyses guidelines in reporting the included studies’ results. The cluster mapping approach was used to identify and classify the barriers into themes.

**Results:**

Thirteen eligible studies were included in this current review. Seven of the thirteen studies used quantitative cross-sectional research design, while six used qualitative cross-sectional research design. These studies were conducted across the United Kingdom. Five themes were developed from the cluster mapping, and thirty-four sub-theme barriers to the uptake of breast and cervical cancer screening among Black, Asian, and Minority Ethnic women in the United Kingdom were identified. The developed themes in relation to the barriers include; socio-demographic characteristics, health service delivery, cultural, religious & language, the gap in knowledge & awareness, and emotional, sexual & family support.

**Conclusion:**

The study concluded that barriers in socio-demographic characteristics, health service delivery, cultural, religious and language, the gap in knowledge & awareness, and emotional, sexual & family support were identified as non-uptake of breast and cervical cancer screening among Black, Asian, and Minority Ethnic women in the United Kingdom. Reducing or eliminating these barriers would improve the benefits of timely breast and cervical cancer screening in the United Kingdom.

**Supplementary Information:**

The online version contains supplementary material available at 10.1186/s12913-023-09410-x.

## Background

Cancer is currently the leading cause of mortality globally, with new cancer cases estimated at 19.3 million and almost 10 million deaths in 2020 [1–3]. These adverse statistics on cancer have serious implications for global public health, life expectancy, and labour force participation [2, 4–6]. Breast cancer was the leading cause of cancer incidence, contributing an estimated 2.3 million cases to the global cancer incidence in 2020; this contribution represents 11.7% of cancer worldwide [1]. Breast cancer incidence and mortality prevalence among women varies from region to region and is more prominent in the European region than in other regions [1, 7, 8]. The disparities in the distribution of breast cancer incidence and mortality among women in Europe have been linked to late diagnosis, preventing early detection and treatment, and leading to low survivor rates [5, 9]. Cervical cancer was reported as the fourth most frequently diagnosed and leading cause of cancer mortality among women globally, with about 604,000 new cases and 342,000 deaths in the year 2020 [1]. Although cervical cancer incidence and mortality prevalence rates are not high in the European region and other high-income countries, however, studies have recently raised concern about the rise in cervical cancer among immigrant women in Europe, leading to apparent health inequalities [10–13].

The United Kingdom has been identified as one of the regions with high rates of breast and cervical cancer incidence, morbidity, and mortality among women, which is attributed largely to inequality in the uptake of prescribed breast and cervical screenings [1]. The English National Breast and Cervical programmes in the United Kingdom were saddled with the responsibility of preventing cancer by treating precancerous changes or ensuring diagnosis at the early stages when treatment outcomes are more successful [14, 15]. Besides the English National Breast and Cervical programmes mandates, the Department of Health's cancer outcome strategy since 2011 has made it its main focus to promote cancer screenings, particularly breast and cervical cancer, in order to increase early diagnosis and save lives [15–17].

Additionally, to ensure the high rate of breast and cervical cancer-related morbidity and mortality are reduced, the government introduced guidelines for an automatic invite for breast cancer screening uptake for women between the ages 50 to 70 residing in the United Kingdom every three years while women between the age of 25 to 49 years are invited for cervical cancer screening every three years and those between 50 to 64 years are invited every five years [18, 19]. Despite the government's ambitious strategies and plans to reduce the prevalence of incidence and mortality attributable to breast and cervical cancer in the United Kingdom [14–16], about 11,500 and 1,121 women still die yearly from breast and cervical cancer, respectively [18, 20, 21] whilst additional 50,000 and 3,791 women with breast and cervical cancer are diagnosed annually in the United Kingdom [20, 22]. Reducing these rates depends largely on women's participation in breast and cervical cancer screenings in the United Kingdom [23].

However, participating in the United Kingdom cancers screening programmes is influenced by several barriers, which are more prominent among the minority or underrepresented women population, often referred to as Black, Asian, and Minority Ethnic groups, and these barriers continue to contribute to the high prevalence rate of breast and cervical cancers among women in the United Kingdom [23–25]. Accelerating the uptake of these screenings would require identifying the barriers influencing the non-utilisation of these screening services and providing requisites programmes and plans to overcome the barriers [26–28]. Nevertheless, previous studies conducted on the barriers to the uptake of breast and cervical cancer screenings among Black, Asian, and Minority Ethnic group women in the United Kingdom using either primary or secondary data source; however, to the best of our knowledge, no study has been able to synthesise all the barriers for the last 12 years using a systematic review [29, 30].

Overcoming the barriers in the non-uptake of breast and cervical cancer screening among the Black, Asian, and Minority Ethnic group women in the United Kingdom requires collating all available evidence on barriers preventing the uptake of the screening, and this may be used to develop necessary interventions that may help with early detection and treatment, improved health outcomes, and ultimately accelerate the achievement of sustainable development goal 3, which seeks to ensure healthy lives and promote the well-being of all at all ages by the year 2030 [31]. Thus, to address this, a mixed-methods systematic review was utilised toidentify the barriers to breast and cervical cancer screening uptake among Black, Asian, and Minority Ethnic women in the United Kingdom between January 2010 to July 2022 with a research question of ‘What are the barriers to the uptake of breast and cervical cancer screening among Black, Asian, and Minority Ethnic women in the United Kingdom?’.

## Methods

This review was systematically conducted in accordance with the 2015 and 2020 Joanna Briggs Institute (JBI) methodology guidelines for mixed-methods systematic reviews [32, 33], and reported the findings following the Preferred Reporting Items for Systematic Reviews and Meta-Analyses (PRISMA) [34]. A mixed-method systematic review is essential in providing unique insights into challenges around healthcare service delivery [33]. This mixed-method systematic review was registered with the Prospero registration number CRD42022381510.

### Data source and search strategy

The search terms were developed strategically and cross-checked by both authors (OAB & NH). To ensure that the search terms and strategy were without bias and comprehensive, a preliminary search of both Medical Literature Analysis and Retrieval System Online (MEDLINE) and Cumulative Index to Nursing and Allied Health Literature (CINAHL) was undertaken by scrutinising the text words in the title, abstract, and the index terms used to describe the article which is often known as keywords. The initial search showed the available literature and identified appropriate search terms for subsequent searches. The preliminary search returns a large number of relevant articles, which indicates a robust search. These same search terms were used to search other electronic databases included in this study; it is recommended that search strategies are not restricted to English, given the resources available in other languages [35]. However, this study only included articles published in English between January 2010 and July 2022 (Appendix III). Only English language studies were considered because both authors are unable to read and write in other languages other than the English language, and only studies between 2010 to 2022 were considered because, according to Cancer Research UK, there was an increase of 11.4% and 7.7% in the number of new cases for both breast and cervical cancers respectively in the United Kingdom between 2010 to 2021 [36].

### Electronic database search

Following the preliminary search of relevant articles from MEDLINE and CINAHL databases using relevant search terms and strategies, the same search terms and strategies were adopted for other databases searches including the British Nursing Index (BNI), Web of Science (WOS), EMBASE, and Scopus to ensure consistency of the process. This search returns numerous relevant articles. This systematic review also applied another method in retrieving relevant articles by checking the referencing list of the included articles to include additional relevant articles. Two articles were retrieved as additional to the eligible articles in this review.

The Population, Intervention, Context, Outcome, Timing and Study type (PICOTS) template components align with the review research questions. The PICOTS table was applied to develop and review the inclusion and exclusion criteria (Table 1).

**Table 1:** The components of the PICOTS template

Study Component	Criteria
Population	Black, Asian, minority, and Ethnic women of any age
Intervention	Uptake of breast and cervical cancer screening
Context	United Kingdom
Outcome	Barriers
Timing	January 2010 to July 2022
Study type	Quantitative, qualitative, mixed-method, and published in English

### Inclusion and exclusion criteria

The PICOTS table included above was applied to developing and defining the review inclusion and exclusion criteria. According to the JBI [37], the inclusion and exclusion criteria must be clearly stated from the initial review stage to ensure that relevant articles are included as eligible articles whilst minimising the selection bias risk. Consequently, PICOTS was fully utilised whilst searching and screening for eligible studies. Table 2 below shows the inclusion and exclusion criteria.

**Table 2:** The inclusion and exclusion criteria

PICOTS	Inclusion	Exclusion
Population	Black, Asian, minority, and Ethnic women of any age	White Ethnic and majority group women only
Intervention	Uptake of breast and cervical cancer screening	-Treatment of breast and cervical cancer -Diagnosis of breast and cervical cancer -Uptake of other forms of cancer screenings
Context	-United Kingdom -England -Northern Ireland -Scotland -Wales	-Other European countries -Other high-income countries -Low-and-middle-income countries
Outcome	-Barriers to breast and cervical cancer screening uptake	-Barriers associated with the treatment of breast and cervical cancer -Barriers associated with the diagnosis of breast and cervical cancer -Barriers associated with other forms of cancer
Timing	-January 2010 to July 2022	-Before January 2010 -After July 2022
Study type	-Quantitative -Qualitative -Mixed method -English language	-Secondary review studies -Unpublished -Other languages -Editorials and commentaries

### Study selection

The title and abstracts were sifted by importing all the retrieved articles into RefWorks and eliminating duplicates. Then the remaining article's title and abstract were checked against the inclusion and exclusion criteria to identify potentially relevant studies. All articles with limited information in the abstracts were included for full-text reading in the second phase of the shifting. Detailed information about the study selection is provided in the result section of this review using the PRISMA flowchart. The selection of eligible studies was completed transparently by ensuring that all the processes involved in the study selection were well documented at every step, as recommended by Page [34].

The second stage of the study selection process was full-text sifting. All the articles were eligible for full-text screening after the title and abstract sifted were downloaded for reading. The article included for full-text screening that was unavailable as open access on the website was accessed using Canterbury Christ Church University’s library. Reading and selecting eligible studies were confirmed against the inclusion and exclusion criteria; some studies that required further reading were read more than once to ensure their suitability before inclusion.

### Data extraction and synthesis

The extraction of relevant data was done twice to ensure no important information was left unextracted. The extraction of relevant data was based on specific details about the study of interest (Breast cancer, cervical screening, barriers associated with the uptake, Black Asian and Minority Ethnic women, United Kingdom), the method utilised in the study, and the significance of the outcome variable. Only data on Black Asian and Minority Ethnic women were extracted for articles that included other population groups of women on breast and cervical cancer screening uptake in the United Kingdom [35]. To generate the quantitative evidence from the eligible studies a significant relationship of barriers associated with the uptake of breast and cervical cancer among Black Asian and Minority Ethnic women was considered, while direct quotes provided by the respondents in the eligible studies were extracted for qualitative evidence [33].

### Assessment of methodological quality

[38] After selecting articles that met the inclusion criteria, a quality assessment of 13 published articles was conducted. Porritt [39] argued that it is important to conduct a quality assessment of eligible articles for a review because low-quality articles may affect the review's credibility. To reduce the risk of bias and ensure that the studies included in this review were all high-quality. The Mixed Methods Appraisal Tool (MMAT) is often utilised to evaluate and appraise qualitative, quantitative, or mixed-methods research designs. This tool assessed the eligible articles included in this review since qualitative and quantitative studies were included [40]. The MMAT assesses the appropriateness of the study aim and design, participant recruitment, adequacy and methodology, data collection, presentation of findings, data analysis, authors’ discussions, and conclusions. Both authors independently reviewed the eligible articles and assigned a quality rating. There were no discrepancies between the two reviewers (OAB & NH) regarding the quality assessment of the articles included in this study [40, 41]. The eligible articles were appraised based on six methodological quality criteria: research questions, representativeness of the target population, rate of non-response, research measurement, and how the research questions were analysed [40]. All the scores were summed up, and no article was dropped because the least score was 80% [40], as shown in Appendix 1.

### Data analysis and emerging clusters

This review utilised the emerging clusters approach in synthesising the data extracted from the included studies [42–44]. The analysis process was in two stages. The first stage involves the identification of all barriers to breast and cervical cancer screening uptake among Black Asian and Minority Ethnic women in the United Kingdom in the included studies. The second stage involved structuring and sorting those identified barriers into clusters; this is the clustering of all the barriers in each study according to how they relate to each other leading to the identification of 5 clusters and 34 barriers [43]. The concept of the cluster mapping approach seems sufficient since the study aims to identify the barriers associated with the uptake of breast and cervical cancer screening among Black Asian and Minority Ethnic women in the United Kingdom. Therefore, the findings were presented in narrative form using tables [45]. To generate the quantitative evidence from the eligible studies a significant relationship of barriers associated with the uptake of breast and cervical cancer among Black Asian and Minority Ethnic women was considered for quantitative studies, while direct quotes provided by the respondents in the eligible studies were extracted for qualitative studiesevidence [33]. Then, a detailed analysis of findings extracted from the included eligible articles was achieved by using a cluster approach; similar content was clustered into five categories as themes [44]. The organisation, assessments, and data analysis are important because the analysis of the data extracted from the included studies should answer the study’s research questions [46, 47]. These clusterings were developed through in-depth reading and interpretation of the included articles’ results section. This increased the in-depth knowledge of the authors in the study, which improved the quality of the results presented in this study [44, 48, 49].

## Results

**Fig. 1:**
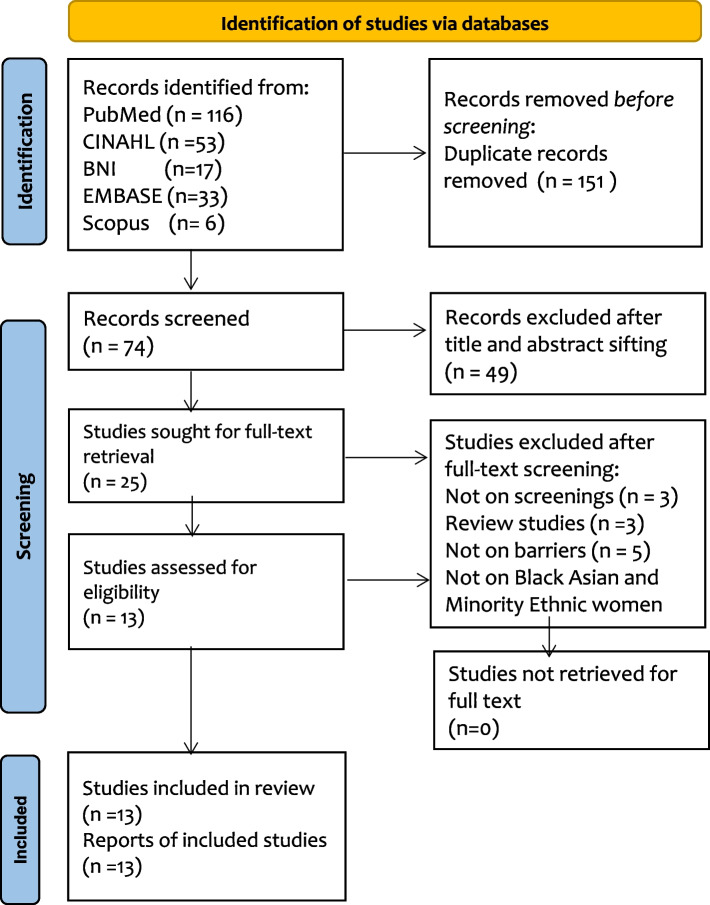
Fig. 1 PRISMA 2020 Flow diagram [34]

An initial search yielded a total of 225 studies identified from the included databases. One hundred fifty-one studies were excluded as duplicates from the search results, whilst additional 49 studies were excluded after the title, and abstract sifting of the studies does not fit into the inclusion criteria of this review, leaving 25 studies for studies for full-text sought and screening. All 25 full-text studies were retrieved, and 12 studies did not meet the study inclusion criteria of reporting barriers to breast cancer screening among Black Asian and Minority Ethnic in the United Kingdom. The 12 full-text studies screened that did not meet the review criteria were based on the following; 3 studies did not report screening [50–52], 3 studies were previously published review studies [53–56], 5 studies did not state any barriers [57–60], and 1 study was not on Black Asian and Minority Ethnic women [61] as shown in appendix II. 13 studies that met the inclusion criteria were finally included in the review for analysis, as shown in Fig. 1.

To ensure that the eligible included studies were good qualities, the MMAT was used to appraise the eligible articles based on six methodological quality criteria: research questions, representativeness of the target population, rate of non-response, research measurement, and how the research questions were analysed [40]. All the scores were summed up, and no article was dropped because the least score was 80%, as shown in Appendix 1.

### Characteristics of included studies

The characteristics of the 13 studies included in the systematic review, such as the study aim, country where the research was conducted, study design, and participant characteristics, were illustrated in Table 3. In relation to this study focus, out of the 13 studies in this review, 8 studies focused on the range of barriers to breast cancer screening among Black Asian and Minority Ethnic women in the United Kingdom [62–69], whilst 5 studies focused on the barriers to cervical cancer screening among Black Asian and Minority Ethnic women in the United Kingdom [29, 30, 70–72].

**Table 3:** Characteristics and results of the included studies

First Author (year)	Country	Study aim	Study design	Participants characteristics	Key findings	
					Barriers to Breast cancer screening	Barriers to Cervical cancer screening
Renshaw (2010) [68]	England	To produce estimates of the breast cancer screening attendance for women in White, Black, and Asian Ethnic groups, while considering other factors, such as socioeconomic and age group	Quantitative cross-sectional study	742,786 women between the ages of 50 to 70 invited for NHS breast cancer screening	- Low socioeconomic status - Invitation type (First call and routine recall)	N/A
Jain (2012) [66]	United Kingdom	To assess how Breast Screening Units in the United Kingdom communicate with their South Asian screening population	Quantitative cross-sectional study	66 units attended the survey; 56 units from England, 3 from Scotland, 1 from Wales, and 1 from Northern Ireland 4 units were anonymous	- Language and communication - Lack of medical knowledge from interpreters - Short appointment time - Lack of cultural competence from health professionals	N/A
Woof (2020) [69]	England	To explore the experiences of British-Pakistani women when accessing the NHS Breast Screening Programme	Qualitative cross-sectional study	19 one-to-one interviews were conducted among British-Pakistani women in East Lancashire, United Kingdom	- Limited/no literacy level - Lack of knowledge of breast cancer/screening service procedure - Incomprehensible terminologies - Cultural beliefs - Symptomatic service	N/A
Bansal (2012) [64]	Scotland	To investigate variation in the uptake of breast cancer screening in minority ethnic groups	A quantitative cross-sectional study	139,374 women were invited for breast screening uptake in Scotland	- Educational level	N/A
Forbes (2011) [65]	England	To examine ethnic differences in breast cancer awareness and barriers to symptomatic presentation in East London	Quantitative cross-sectional population survey	1515 women who were aged 30 and above living in East London	- Embarrassment and fear - Cultural differences - Lack of knowledge	N/A
Karbani (2011) [67]	United Kingdom	To explore attitudes, knowledge, and understanding of breast cancer and preventive measures amongst South Asian women who were breast cancer patients	Qualitative cross-sectional study	24 South Asian breast cancer women were interviewed in the United Kingdom	- Poor knowledge and awareness of the breast screening program - Language barrier	N/A
Bamidele (2017) [62]	England	To determine factors associated with low uptake of breast screening among Black African women living in Luton	Qualitative cross-sectional study	6 focus group discussions were conducted among 25 Black African women aged 35 to 70 residing in Luton	- Cultural belief on cancer sigma - Lack of knowledge - Timing of the appointment - Spiritual belief - Anxiety and fatalism	N/A
Banning (2011) [63]	United Kingdom	To explore the views and practices of black women on breast screening	Qualitative cross-sectional study	Snowball sampling was used to recruit 10 Black British women that have resided in the United Kingdom for not less than 5 years	- Lack of family support - Cost of travelling & other expenses - Negative and unhelpful attitude of healthcare staff - Lack of knowledge	N/A
Marlow (2017) [30]	United Kingdom	- To establish the percentage of British women classified in each cervical cancer screening non-participant type - To identify sociodemographic correlates with each nonparticipant type	Quantitative cross-sectional population survey	Data were collected using face-to-face computer-assisted personal interviews (CAPIs) among 3,661 women across the United Kingdom	N/A	- Lack of awareness
Marlow (2015a) [71]	England	To explore self-perceived barriers to cervical screening attendance among ethnic minority women compared to white British women	Qualitative interview study	43 ethnically diverse women in London Boroughs were interviewed using a semi-structured interview schedule	N/A	- Lack of awareness - Lack of knowledge about medical terminologies - Emotional barriers such as fear, embarrassment, and shame - Health workers’ attitude - Distance to healthcare centers - Absence of symptoms
Ekechi (2014) [70]	England	To examine the socio-demographic and ethnicity-related predictors of cervical cancer knowledge, cervical screening attendance and reasons for non-attendance among Black women in London	Quantitative cross-sectional	652 eligible women were interviewed using a questionnaire in 59 salons across London	N/A	- Younger and unmarried women - Religious services attendance - Absence of symptoms - Cost and Logistics of travelling to health care centers - Fear of test procedure
Marlow (2015b) [72]	England	To examine the socio-demographic and attitudinal correlates of cervical screening non-attendance among Black Asian and Minority Ethnic women	Quantitative cross-sectional	720 Black Asian and Minority Ethnic women between the age of 30 to 60 years across England were interviewed through the ethnic focus platform	N/A	- Older women (aged 51–60 years) - Migrants women - Language and communication - Absence of symptoms - Sexual inactivity - Logistics challenges in attending the healthcare services
Nelson ( 2021) [29]	Scotland	To explore experiences of cervical screening participation and non-participation of women from minority ethnic populations in Scotland	Qualitative comparison group study	50 Black Asian and Minority Ethnic women between the age of 30–65 were interviewed across Scotland	N/A	- Emotional barriers such as fear, dread, or nervousness - Limited appointment time and other commitments such as work and care responsibilities - Not sexually active - Fear of racism, feeling ashamed - Ignorance of what screening is all about

### Country

England was the highest country with number of studies with a total of 7 studies; 4 studies were conducted on barriers to breast cancer screening, whilst 3 studies were conducted on barriers to cervical cancer screening between January 2010 to July 2022 [30, 62, 65, 68–71]. Four studies were conducted in the United Kingdom, three on barriers to breast cancer screening and only 1 study was on cervical cancer screening [30, 63, 66, 67]. The country with the least studies was Scotland, with only 2 studies [29, 64].

### Study design

Out of the 13 included studies, 7 used a quantitative cross-sectional study design [30, 64–66, 68, 70, 71], while 6 used a qualitative cross-sectional study design [29, 62, 63, 67, 69, 72].

### Participants characteristics

There were variations in respondents interviewed in the 13 studies included in the review. The variation in the characteristics of the respondents ranges from the age of the respondents to the study sample sizes and year of residency [29, 30, 62–72]. However, all studies were conducted on Black Asian and Minority Ethnic women in the United Kingdom who can give information on barriers to either breast cancer or cervical cancer screening (Table 3).

### Key findings in themes on barriers to breast and cervical cancer screening

Five themes were developed as barriers to breast and cervical cancer screening among Black Asian and Minority Ethnic women in the United Kingdom between January 2010 to July 2022. Thirty-four barriers (key findings) were identified from the 13 included studies; breast and cervical cancer screenings have 17 barriers each, and these barriers were categorised under five themes discussed below.

### Theme 1: Socio-demographic-related barriers

Out of the 13 included studies in this review, 6 studies [63, 64, 68–70, 72] reported barriers related to socio-demographic factors for breast and cervical cancer screening. The barriers reported include; low socioeconomic status, educational level (Limited/no literacy), cost of travelling & other expenses for breast cancer, whilst the barriers reported for cervical cancer were older aged women (51 to 60 years), migrant women, younger and unmarried women, cost, and logistics of travelling as shown in Table 4.

**Table 4:** Summary of the results in themes

Themes	Barriers to screenings		Studies
	Breast cancer	Cervical cancer	
Socio-demographic related barriers	- Low socioeconomic status - Educational level (Limited/no literacy) - Cost of travelling & other expenses	- Older aged women (51 to 60 years) - Migrant women - Younger and unmarried women - Cost and logistics of travelling	Renshaw [68]; Bansal [64]; Marlow [72]; Woof [69] Banning [63]; Ekechi [70]
Healthcare service delivery-related barriers	- Invitation types - Interpreters lack medical knowledge - Short/lack of appointment time - Health professionals lack cultural competence - Negative/unhelpful attitude of health workers	- Health workers' negative attitude - Distance to healthcare centers - Limited/lack of appointment time	Renshaw [68]; Jain [66]; Bamidele [62]; Banning [63]; Marlow [71]; Nelson [29]
Cultural, religious, and language-related barriers	- Language and communication - Cultural beliefs/differences on cancer sigma - Spiritual belief	- Religious belief - Language and communication	Jain [66]; Forbes [65]; Karbani [67]; Bamidele [62]; Ekechi [70]; Marlow [72]
Gaps in knowledge and awareness-related barriers	- Lack of knowledge of breast screening services and procedures - Incomprehensible medical terminologies - Absence of symptoms	- Lack of knowledge and awareness of cervical cancer screening - Lack of knowledge about medical terminologies - Absence of symptoms	Woof [69]; Forbes [65]; Karbani [67]; Bamidele [62]; Banning [63]; Marlow [30]; Marlow [71]; Ekechi [70]; Nelson [29]
Emotional, sexual & family support-related barriers	- Embarrassment and fear - Anxiety and fatalism - Lack of family support	- Embarrassment, fear, and shame - Fear of test procedure - Sexual inactivity - Work and family responsibilities - Fear of racism	Forbes [65]; Bamidele [62]; Banning [63]; Marlow [71]; Ekechi [70]; Marlow [72]; Nelson [29]

### Theme 2: Health service delivery-related barriers

Six studies [29, 62, 63, 66, 68, 71] out of the 13 included studies reported barriers around health service delivery to non-uptake of breast and cervical cancer screening. For breast cancer screening, barriers reported were invitation types, interpreter lack of medical knowledge, short/lack of appointment time, health professionals lack of cultural competence, and negative/unhelpful attitude of health workers, whilst barriers reported for cervical screening uptake were health workers negative attitude, distance to healthcare centres, limited/lack of appointment time as shown in Table 4.

### Theme 3: Cultural, religious, and language-related barriers

Barriers relating to culture, religion and language were reported by 7 studies [62, 65–67, 70, 72] out of the 13 included studies. Language and communication, cultural beliefs/differences on cancer sigma, and spiritual belief were the barriers reported to the uptake of breast cancer among Black Asian and Minority Ethnic women in the United Kingdom, whilst barriers such as religious belief and language & communication were reported for cervical cancer screening uptake as shown in Table 4

### Theme 4: Gap in knowledge and awareness-related barriers

Out of the 13 studies included, 10 studies [29, 30, 62, 63, 65, 67, 69–72] reported a gap in knowledge and awareness-related factors as barriers to breast and cervical cancer screening among Black Asian and Minority Ethnic women in the United Kingdom. Barriers related to breast cancer include; lack of knowledge of breast screening services & procedures, incomprehensible medical terminologies, and absence of symptoms, whilst barriers related to cervical cancer include; lack of knowledge and awareness of cervical cancer screening, lack of knowledge about medical terminologies, and absence of symptoms as shown in Table 4.

### Theme 5: Emotional, sexual and family support-related barriers

Seven studies [29, 62, 63, 65, 70–72] of the 13 included studies reported barriers relating to emotional, sexual & family support as the reason for the non-uptake of breast and cervical cancer screening. Breast screening barriers include embarrassment & fear, anxiety & fatalism, and lack of family support, whilst barriers reported for cervical screening include embarrassment, fear & shame, fear of test procedure, sexual inactivity, work & family responsibilities, and fear of racism, as shown in Table 4.

## Discussion

The studies included in this mixed-methods systematic review was 13, presenting a wide range of barriers to breast cancer and cervical cancer screening uptake among Black Asian and Minority Ethnic women in the United Kingdom between January 2010 to July 2022. Although the studies included two important NCD outcome variables and were conducted in different regions within the United Kingdom, the barriers highlighted by the studies were consistent throughout the study locations in the United Kingdom. The mixed-methods systematic review extracted 34 key findings and developed 5 themes from similar key findings (clusters) from the 13 included studies.

Five synthesised findings generated in this review in relation to barriers to breast cancer and cervical cancer screening uptake among Black Asian and Minority Ethnic women in the United Kingdom were socio-demographic-related barriers, healthcare service delivery-related barriers, cultural, religious & language-related barriers, the gap in knowledge & awareness-related barriers, and emotional, and sexual & family support related barriers.

### Socio-demographic related barriers

Both breast and cervical cancers are preventable diseases, provided that women of reproductive ages adhere to regular screening, which previous studies have reportedly recommended to facilitate early detection and influence better treatment outcomes for these two types of cancers [73–76]. Despite the evidence supporting the effectiveness of regular screening for both diseases, other evidence counteractively shows that women of reproductive age often face socio-demographic-related barriers preventing them from adhering to regular screening practices [77, 78].

The findings from this study ascertained the point raised above as nearly half of the studies assessed in this review specifically presented the socio-demographic-related barriers as one of the categories of barriers preventing Black Asian and Minority Ethnic women in the United Kingdom from accessing breast and cervical cancer screening services. The three key socio-demographic-related barriers highlighted for breast cancer screening include the low socioeconomic status of Black Asian and Minority Ethnic women, which is likely to be a result of women taking more household responsibilities as primary caregivers to their children which often predisposed them to some socioeconomic disadvantages like inability to be gainfully employed overtime, leading to financial independence to seek medical attention as at when due [79, 80]. Globally, the high cost of care has been reported as an important barrier preventing women from accessing screening services, especially for migrant women with no stable jobs, and this is another way in which the socioeconomic status of women hinders their ability to access breast and cervical cancer screening services [80].

The two other barriers revealed in this review among Black Asian and Minority Ethnic women were also linked to low socioeconomic status because of the role that socioeconomic status plays in the attainment of educational qualifications and the ability/inability to afford the cost of medical and non-medical expenses among socioeconomically disadvantaged people. Several other studies [81–83] conducted among women from other continents across the world identified similar socio-demographic barriers preventing women from accessing breast and cervical cancer screening services, and these studies supported the current study's findings. For instance, Park's [81] study among Korean women showed that socio-demographic factors like marital status, education, residency and health insurance status often predict the categories of women who uptake breast and cervical cancer screening and that women of higher socioeconomic status uptake these services more compared to women of lower socioeconomic status. Although, there are some variations in the specific socio-demographic barriers listed in this current review and Park’s [81] study, such as the health insurance status, marital status and residency that were not identified in the current review.

### Health service delivery-related barriers

In some cases, women often overcome sociodemographic barriers to breast and cervical cancer screening but meet a brick wall at the facility where they intend to uptake the screening services [82]. These barriers often destroy women’s resolve to seek healthcare services because healthcare services deliver-related barriers often discourage them from requesting screening services [83]. The findings from this research showed that Black Asian and Minority Ethnic women encountered several other barriers related to healthcare service provision in their quest to access screening services for breast and cervical cancer. Adunlin’s study [84] reported similar findings to the current review on some of the healthcare-related barriers influencing migrant women’s breast and cervical cancer screening, and these include the knowledge gap between the medical personnel and the care seekers, which stems from either interpreters’ ability to pass accurate information to the migrant women where language barriers are predominantly existing. This aspect of Adunlin's [84] study coincides with the findings from this review and other systematic and scoping reviews like the ones conducted by de Cuevas [85], Ferdous [86], and Boom [87]. All these three studies highlighted the role of healthcare-system-related barriers in preventing ethnic minoritised women from attending breast and cervical cancer screening.

In some studies with similar findings to this current review, like those of Chidyaonga-Maseko [88], Gele [89], Orji and Yamashita [90], other factors like cultural differences and fear of racial discrimination were identified as major push and pull factors influencing healthcare-related barriers. Marques [91]'s research on immigrant women in Europe revealed findings that followed a similar pattern to those of the current review by linking the healthcare system barriers to some inherent factors that are peculiar to ethnic minority women across Europe (like Black Asian and Minority Ethnic women in the United Kingdom).

Previously experienced healthcare services disparities from racial and Ethnic group maybe linked to the reasons for not seeking healthcare services [90, 92–94]. All these factors may contribute to the healthcare-system-related barriers [92].

### Cultural, religious & language related barriers

The acculturation process has been reported to influence healthcare-seeking behaviour as it takes time for different categories of people at different times and for different reasons because of the rigid nature of their inherent behaviour tied to their cultural and religious beliefs from their country of origin [95]. Language, culture, and religious beliefs, in the case of Black Asian and Minority Ethnic in the United Kingdom, are not only a barrier to health-seeking behaviour but other social and cooperative engagement like employability, social engagement, women in manufacturing and construction industries, which also ping back to their ability to access healthcare services like breast and cervical cancer screening [96]. The findings from this review show that religious beliefs, culture, and language also contribute to the barriers to taking up breast cancer and cervical cancer screening as there are possibilities that Black Asian and Minority Ethnic women in the United Kingdom have reluctancy to give up their inherent belief about their health and these cannot be easily corrected when trying to get incorporated into an entirely new system [97].

Marques's [91] study conducted among minoritised women in Europe showed a similar pattern of findings to the current review, as their study listed cultural differences as the key barriers preventing these women from accessing cervical cancer screening services. Although, their study also reported other barriers like the healthcare system-related barrier and the cultural differences, which are all accounted for in this current review findings.

These barriers could be attributed to the inability to understand or communicate due to language differences can also influence poor uptake of cancer screening procedures for breast and cervical cancer [91]. Spiritual and religious beliefs were also identified as one of the barriers to taking up cancer screening. Spiritual and religious beliefs such that many Black Asian and Minority Ethnic communities will rather request herbs to be sent from their native country than visit a healthcare facility, and the belief in spiritual healing was also identified as barriers in this review such that the Black Asian and Minority Ethnic community prefer to believe in the supernatural intervention than to seek medical advice [91, 97]. Some believe that talking about cancer could be a form of affirmation, resulting in them attracting and developing it; therefore, they will not discuss it. Cultural belief is also a barrier to screening uptake; one of these beliefs is having sexual relationships outside of marriage and the possibility that a cervical cancer diagnosis would be seen as embarrassing by certain women [97].

These findings were similar to what has been reported by other studies that highlighted that wizardry, sexual relationships with multiple partners, and the use of herbs through the vagina as the cause of cervical cancer, while the necessity for spousal consent, discrimination at hospitals, lack of awareness, religious and cultural responsibilities of modesty, the gender of healthcare personnel, fear of nosocomial infections, fear of disclosure of results, and fear of publication of results as barriers to screening were the barriers reported in their studies [96, 97]. Similarly, Kirubarajan's [98] systematic review also identified cultural beliefs and language barriers to cervical cancer screening uptake. Another systematic review among South Asian women by de Cuevas [85] on breast and cervical cancer screening uptake also found cultural practices as barriers. Mafiana [99] also reported traditional, cultural and religious beliefs are some of the barriers to screening.

### The gap in knowledge & awareness-related barriers

Knowledge and awareness are vital in influencing health-seeking behaviour, especially among women who may be dispositioned for one reason or the other, as in the case of Black Asian and Minority Ethnic women in the United Kingdom [100]. Findings from this review show that knowledge and awareness-related barriers influence non-uptake of breast and cervical screening practices among Black Asian and Minority Ethnic women due to their inability to seek health services and make informed decisions as a result of probable poor knowledge about where to seek proper healthcare services, including how and why to seek healthcare services and other information surrounding breast and cervical screening practices.

Several studies conducted among women across the globe identified knowledge and awareness as factors hindering healthcare services among Black Asian and Minority Ethnic women [86, 101–103]. A study conducted by Sultana [103] on “Awareness About Cervical Cancer in Pakistani Women” shows that the major barrier to cervical screening is a lack of awareness and fallacy towards the screening, which correlates with the current review. However, the study identified the screening fallacy as a barrier, which dissociates from the current review. In another study conducted by Moodley [76] among sub-Saharan African women in South Africa and Uganda, it was ascertained that breast cancer awareness influences healthcare practices. However, the study scope targeted married women living with their partners, married women not living with their partners, urban dwellers, and rural dwellers, which were not identified in the current review.

More so, research conducted in some European countries has shown that women participating in breast and cervical cancer screening examinations have lower mortality tendency,however just a few usually undergo screening due to a knowledge gap [7, 91]. This was also true in other continents; for instance, a study conducted among minoritised women residing in Australia by Alam [104] who concluded that insufficient knowledge is one of the major barriers to cervical cancer screening uptake among this group of women. However, some of the studies included obese women, which does not correlate with the current review [73, 91, 105]. Research conducted among Ethnic minority backgrounds in the United Kingdom shows that inadequate awareness and knowledge about cervical screening and related tests in Ethnic minority communities increases the cervical cancer mortality rate [71]. The study ascertained the gap in knowledge and awareness identified in this current review.

### Emotional, sexual, and family support-related barriers

Support from family is crucial to managing any health condition, especially cancer management, which usually over some time and can be draining. Emotional and family support ensures that the individual is not alone in the fight [106]. This review found that lack of family and emotional support, embarrassment, fear and shame, and fear of the test procedure are major barriers to the uptake of cervical and breast cancer screening. It has been revealed from the findings of this review that emotional support provided by family and friends could encourage the uptake of breast and cervical cancer screening.

This aspect of the findings is supported by Molina's [107] study on the role of family and social interactions in breast cancer screening among women of Latina origin. The point of concordance in the two studies [107] and this current review was on the importance of family support and recommendations in the utilisation of healthcare screening services and without, which may form a significant emotional barrier for women from ethnic minoritised groups that were the focus of the current review. Similarly, Adegboyega's [108] study reported slightly similar findings about those barriers related to family support with special reference to spousal approval, which is a key decider in women’s healthcare-seeking behaviour among immigrants from sub-Saharan Africa with its engraved patriarchal social structure in marital relations.

Similarly, this review also showed that many Black Asian and Minority Ethnic women often feel embarrassed when it comes to testing procedures, and they may also be afraid of being treated differently because of their skin colour or race. The issue of racial disparities as an emotional barrier to the uptake of breast and cervical screening services was documented in other studies like that of White-Means [94], Orji and Yamashita [90], where women of African descent and other Ethnic minorities in the United States of America often feel stigmatised for their skin colour and race in all ramifications and this introduced emotional barriers that prevent this category of women from seeking healthcare services related to breast and cervical cancer screening.

Findings from this study also showed that the fear of the test procedure being perceived as painful and uncomfortable has resulted in many Black Asian and Minority Ethnic women withdrawing from the screening procedure. It was also found that Black Asian and Minority Ethnic women, especially the older ones, often express their bodies as private; hence they will feel embarrassed taking the test. It was also observed that the perceived shame, with the main driver being what people would say, how they developed cancer, was a limiting factor to taking the screening test. They feel it is better not to know than for it to become a stigma. In Nyblade's [109] study, the Indian community discovered that seeking screening, early diagnosis, or treatment was not advised among women because of the stigma associated with receiving a cancer diagnosis, and Ginjupalli [110] corroborated this; in the same vein, Momenimovahed [111] identified pain and embarrassment as barriers to screening uptake among their respondents.

### Strengths and limitations

Whale [112] poised that presenting key strengths and limitations of systematic review studies is an important research process that contributes to its credibility to be admitted as evidence by healthcare stakeholders and researchers alike with a perfect understanding of its flaws and thereby make informed decisions based on that. Some of the key strengths identified in this study include its extensive literature search that showed a wide range of primary studies that discussed diverse barriers to breast and cervical cancer screening uptake and solidified the reviewer’s justification for conducting this review. It is vital to note that the strengths identified for this review were as important as its credibility and weaknesses, which will show both the researcher’s lapses and those inherited (from included studies) biases. Another limitation of this review is that the study was only conducted in English; this could have excluded some important studies conducted in other languages.

### Recommendations for future research

This mixed-methods systematic review was conducted to fill some gaps in knowledge and literature about barriers that prevent the uptake of breast cancer and cervical cancer screening among Black Asian and Minority Ethnic women in the United Kingdom, and in doing so, was able to discover other areas for exploration by future research. The barriers were synthesised to form healthcare-system-related and individual-related barriers that hinged on Black Asian and Minority Ethnic women’s emotions, family, knowledge, and sociocultural profiles. However, this study shows that there are gaps in the identification of the most prevalent and significant barriers among the myriads of barriers discussed in this study and therefore recommended that future research should:

- Examine each barrier to link cause and effect using mixed methods research methods (with a meta-analysis approach) to show the most significantly predominant barrier influencing Black Asian and Minority Ethnic women.
- Explore the peculiarities of individual subgroups among Black Asian and Minority Ethnic women to identify barriers unique to a group or those that do not exist in other groups and ascertain whether this is common among Blacks, Asians, or other minority populations. For example, some English-speaking African countries might not have language and interpreter-related barriers. So future research should focus on migrant women of black origin and migrant women of Asian origin.
- Look into each of the barriers, specifically among different groups of women, since this current review has been able to bring together most of the barriers to screening uptake among Black Asian and Minority Ethnic women to link and increase the generalisability of the findings from this current review.

## Conclusion

This review identified barriers to the uptake of breast cancer and cervical cancer screening among Black Asian and Minority Ethnic women in the United Kingdom. The study concluded that barriers were socio-demographic characteristics, healthcare service delivery, cultural, religious & language, the gap in knowledge & awareness, and emotional, sexual & family support. To reduce or eliminate these barriers, continuous campaigns and education on the importance and benefits of timely breast and cervical cancer screening should be made widely available in all public places and hospitals where Black Asian and Minority Ethnic women domicile in the United Kingdom. It also needs to be acknowledged the need for structural changes, including examining public health priorities, exploring political drivers and addressing wider healthcare bureaucracies, as barriers to screening are complex. Tackling health and cancer inequalities requires a variety of public health interventions. Although this can be perceived to be a great deal of work, it has never been more crucial for both research and practice.

## Supplementary Information

**Additional file 1.**

## Data Availability

All data generated and analysed during this study are included in this manuscript as supplementary information.

## Abbreviations

WHO: World Health Organisation
PRISMA: Preferred Reporting Items for Systematic Reviews and Meta-Analyses
MEDLINE: Medical Literature Analysis and Retrieval System Online
CINAHL: Cumulative Index to Nursing and Allied Health Literature
BNI: British Nursing Index
WOS: Web of Science
PICOTS: Population, Intervention, Context, Outcome, Timing and Study type
JBI: Joanna Briggs Institute
MMAT: Mixed Methods Appraisal Tool

## References

[1] Sung H, Ferlay J, Siegel RL, Laversanne M, Soerjomataram I, Jemal A, Bray F: Global cancer statistics 2020: GLOBOCAN estimates of incidence and mortality worldwide for 36 cancers in 185 countries. *CA: a cancer journal for clinicians* 2021, 71:209–249. 10.3322/caac.21660 33538338

[2] World Health Organisation [WHO]: Global health estimates 2015: deaths by cause, age, sex, by country and by region, 2000–2019. In *Geneva: WHO*. pp. 2020; 2020:2020.

[3] Afaya A, Ramazanu S, Bolarinwa OA, Yakong VN, Afaya RA, Aboagye RG, Daniels-Donkor SS, Yahaya A-R, Shin J, Dzomeku VM (2022). Health system barriers influencing timely breast cancer diagnosis and treatment among women in low and middle-income Asian countries: evidence from a mixed-methods systematic review. BMC Health Serv Res.

[4] Bray F, Laversanne M, Weiderpass E, Soerjomataram I (2021). The ever-increasing importance of cancer as a leading cause of premature death worldwide. Cancer.

[5] Mennini FS, Trabucco Aurilio M, Gazzillo S, Nardone C, Sciattella P, Marcellusi A, Migliorini R, Sciannamea V, Piccioni A, Bolcato M (2021). An analysis of the social and economic costs of breast cancer in Italy. Int J Environ Res Public Health.

[6] Carlsen K, Ewertz M, Dalton SO, Badsberg JH, Osler M (2014). Unemployment among breast cancer survivors. Scandinavian journal of public health.

[7] Carioli G, Malvezzi M, Rodriguez T, Bertuccio P, Negri E, La Vecchia C (2017). Trends and predictions to 2020 in breast cancer mortality in Europe. The Breast.

[8] Edet R, Ekundina O, Bolarinwa OA, Babajide J, Nwafor JA (2020). Knowledge of Breast Cancer and Screening Methods among Rural Women in Southwest Nigeria: A Mixed Method Analysis. Advanced Journal of Social Science.

[9] Puliti D, Duffy SW, Miccinesi G, De Koning H, Lynge E, Zappa M, Paci E (2012). Overdiagnosis in mammographic screening for breast cancer in Europe: a literature review. J Med Screen.

[10] Di Napoli A, Ventura M, Grande E, Frova L, Mirisola C, Petrelli A (2022). Nationwide longitudinal population-based study on mortality in Italy by immigrant status. Sci Rep.

[11] Lamminmäki M, Leivonen A, Sarkeala T, Virtanen A, Heinävaara S: Health inequalities among Russian-born immigrant women in Finland: longitudinal analysis on cervical cancer incidence and participation to screening. *Journal of Migration and Health* 2022:100117. 10.1016/j.jmh.2022.100117 PMC9194839 35712528

[12] Haakenstad A, Yearwood JA, Fullman N, Bintz C, Bienhoff K, Weaver MR, Nandakumar V, Joffe JN, LeGrand KE, Knight M (2022). Assessing performance of the Healthcare Access and Quality Index, overall and by select age groups, for 204 countries and territories, 1990–2019: a systematic analysis from the Global Burden of Disease Study 2019. Lancet Glob Health.

[13] Harvey JD, Henrikson HJ, Lu D, Pennini A, Xu R, Ababneh E, Abbasi-Kangevari M, Abbastabar H, Abd-Elsalam SM, Abdoli A: Cancer Incidence, Mortality, Years of Life Lost, Years Lived With Disability, and Disability-Adjusted Life Years for 29 Cancer Groups From 2010 to 2019: A Systematic Analysis for the Global Burden of Disease Study 2019. 2021. 10.1001/jamaoncol.2021.6987 PMC8719276 34967848

[14] Health & Social Care Information Centre [HSCIC]: Cervical Screening Programme, England Statistics for 2012–13. 2014.

[15] Breast Screening Programme, England Statistics for 2011–12. [http://www.hscic.gov.uk/catalogue/PUB13567/bres-scre-prog-eng-2012–13-rep.pdf]

[16] Improving outcomes: a strategy for cancer [https://www.gov.uk/government/publications/the-nationalcancer-strategy]

[17] Weller D, Campbell C (2009). Uptake in cancer screening programmes: a priority in cancer control. Br J Cancer.

[18] Breast cancer information services division [www.isdscotland.org/Health-Topics/Cancer/Cancer-Statistics/Breast/ ]

[19] Uptake for routine breast screening falls - NHS Digital [ https://digital.nhs.uk/news-and-events/news/uptake-for-routine-breast-screeningfalls]

[20] HPV Information centre: United Kingdom; Human Papillomavirus and Related Cancers, Fact Sheet 2021. In *United Kingdom: ICO/IARC Information Centre on HPV and Cancer*; 2021.

[21] Cancer Survival in England - Adults diagnosed, [www.ons.gov.uk/peoplepopulationandcommunity/healthandsocialcare/conditionsanddiseases/datasets/cancersurvivalratescancersurvivalinenglandadultsdiagnosed ]

[22] Cancer registration statistics [www.ons.gov.uk/peoplepopulationandcommunity/healthandsocialcare/conditionsanddiseases /datasets/cancerregistrationstatisticscancerregistrationstatisticsengland]

[23] Hudson S, Brazil D, Teh W, Duffy SW, Myles JP (2016). Effectiveness of timed and non-timed second appointments in improving uptake in breast cancer screening. J Med Screen.

[24] Gianino MM, Lenzi J, Bonaudo M, Fantini MP, Siliquini R, Ricciardi W, Damiani G (2018). Organized screening programmes for breast and cervical cancer in 17 EU countries: trajectories of attendance rates. BMC Public Health.

[25] Stead MJ, Wallis MG, Wheaton ME (1998). Improving uptake in non-attenders of breast screening: selective use of second appointment. J Med Screen.

[26] Marcu A, Marke L, Armes J, Whitaker KL, Ream E: Adapting a breast cancer early presentation intervention for Black women: A focus group study with women of Black African and Black Caribbean descent in the United Kingdom. *European Journal of Cancer Care* 2022:e13652. 10.1111/ecc.13652 PMC9786577 35838142

[27] Ross E, Maguire A, Donnelly M, Mairs A, Hall C, O’Reilly D (2020). Disability as a predictor of breast cancer screening uptake: A population-based study of 57,328 women. J Med Screen.

[28] Tabár L (2021). Chen TH-H, Yen AM-F, Dean PB, Smith RA, Jonsson H, Törnberg S, Chen SL-S, Chiu SY-H, Fann JC-Y: Early detection of breast cancer rectifies inequality of breast cancer outcomes. J Med Screen.

[29] Nelson M, Patton A, Robb K, Weller D, Sheikh A, Ragupathy K, Morrison D, Campbell C (2021). Experiences of cervical screening participation and non-participation in women from minority ethnic populations in Scotland. Health Expect.

[30] Marlow LA, Chorley AJ, Haddrell J, Ferrer R, Waller J (2017). Understanding the heterogeneity of cervical cancer screening non-participants: data from a national sample of British women. Eur J Cancer.

[31] UN: Transforming our world: The 2030 agenda for sustainable development. *New York: United Nations, Department of Economic and Social Affairs* 2015.

[32] Pearson A, White H, Bath-Hextall F, Salmond S, Apostolo J, Kirkpatrick P (2015). A mixed-methods approach to systematic reviews. JBI Evidence Implementation.

[33] Stern C, Lizarondo L, Carrier J, Godfrey C, Rieger K, Salmond S, Apostolo J, Kirkpatrick P, Loveday H (2020). Methodological guidance for the conduct of mixed methods systematic reviews. JBI evidence synthesis.

[34] Page MJ, McKenzie JE, Bossuyt PM, Boutron I, Hoffmann TC, Mulrow CD, Shamseer L, Tetzlaff JM, Akl EA, Brennan SE (2021). The PRISMA 2020 statement: an updated guideline for reporting systematic reviews. BMJ.

[35] McGowan J, Sampson M, Salzwedel DM, Cogo E, Foerster V, Lefebvre C (2016). PRESS peer review of electronic search strategies: 2015 guideline statement. J Clin Epidemiol.

[36] Cancer Statistics [https://www.cancerresearchuk.org/health-professional/cancer-statistics/statistics-by-cancer-type/breast-cancer ]

[37] Systematic Review Resource Package: The Joanna Briggs Institute Method for Systematic Review Research Quick Reference Guide [http://healthindisasters.com/images/Books/Systematic-Review-Resource-Package.pdf]

[38] Grant MJ, Booth A (2009). A typology of reviews: an analysis of 14 review types and associated methodologies. Health Info Libr J.

[39] Porritt K, Gomersall J, Lockwood C (2014). JBI's systematic reviews: study selection and critical appraisal. AJN The American Journal of Nursing.

[40] Hong QN, Pluye P, Fàbregues S, Bartlett G, Boardman F, Cargo M, Dagenais P, Gagnon M-P, Griffiths F, Nicolau B: Mixed methods appraisal tool (MMAT), version 2018. *Registration of copyright* 2018, 1148552.

[41] Pace R, Pluye P, Bartlett G, Macaulay AC, Salsberg J, Jagosh J, Seller R (2012). Testing the reliability and efficiency of the pilot Mixed Methods Appraisal Tool (MMAT) for systematic mixed studies review. Int J Nurs Stud.

[42] Burke JG, O’Campo P, Peak GL, Gielen AC, McDonnell KA, Trochim WM (2005). An introduction to concept mapping as a participatory public health research method. Qual Health Res.

[43] Orsi R (2017). Use of multiple cluster analysis methods to explore the validity of a community outcomes concept map. Eval Program Plann.

[44] Trochim WM (1989). An introduction to concept mapping for planning and evaluation. Eval Program Plann.

[45] Munn Z, Tufanaru C, Aromataris E (2014). JBI's systematic reviews: data extraction and synthesis. AJN The American Journal of Nursing.

[46] Pollock A, Berge E (2018). How to do a systematic review. Int J Stroke.

[47] Renner M, Taylor-Powell E (2003). Analyzing qualitative data.

[48] Auerbach C, Silverstein LB: *Qualitative data: An introduction to coding and analysis.* NYU press; 2003.

[49] Thomson SB: Qualitative research: validity. 2011.

[50] Davies EA, Renshaw C, Dixon S, Møller H, Coupland VH (2013). Socioeconomic and ethnic inequalities in screen-detected breast cancer in London. J Public Health.

[51] Gathani T, Chaudhry A, Chagla L, Chopra S, Copson E, Purushotham A, Vidya R, Cutress R (2021). Ethnicity and breast cancer in the UK: Where are we now?. Eur J Surg Oncol.

[52] Massat NJ, Douglas E, Waller J, Wardle J, Duffy SW (2015). Variation in cervical and breast cancer screening coverage in England: a cross-sectional analysis to characterise districts with atypical behaviour. BMJ Open.

[53] Baird J, Yogeswaran G, Oni G, Wilson E (2021). What can be done to encourage women from Black, Asian and minority ethnic backgrounds to attend breast screening? A qualitative synthesis of barriers and facilitators. Public Health.

[54] Edgar L, Glackin M, Hughes C, Ann Rogers KM (2013). Factors influencing participation in breast cancer screening. British Journal of Nursing.

[55] Vrinten C, Wardle J, Marlow LA (2016). Cancer fear and fatalism among ethnic minority women in the United Kingdom. Br J Cancer.

[56] Woof VG, Ruane H, French DP, Ulph F, Qureshi N, Khan N, Evans DG, Donnelly LS (2020). The introduction of risk stratified screening into the NHS breast screening Programme: views from British-Pakistani women. BMC Cancer.

[57] Brennan M (2017). Breast cancer in ethnic minority groups in developed nations: case studies of the United Kingdom and Australia. Maturitas.

[58] Gorman DR: Breast screening uptake in Polish women in Scotland. *Diversity and Equality in Health and Care* 2016, 13.

[59] Jack RH, Møller H, Robson T, Davies EA (2014). Breast cancer screening uptake among women from different ethnic groups in London: a population-based cohort study. BMJ Open.

[60] Price CL, Szczepura AK, Gumber AK, Patnick J (2010). Comparison of breast and bowel cancer screening uptake patterns in a common cohort of South Asian women in England. BMC Health Serv Res.

[61] Connolly D, Hughes X, Berner A (2020). Barriers and facilitators to cervical cancer screening among transgender men and non-binary people with a cervix: A systematic narrative review. Prev Med.

[62] Bamidele O, Ali N, Papadopoulos C, Randhawa G: Exploring factors contributing to low uptake of the NHS breast cancer screening programme among Black African women in the UK. 2017.

[63] Banning M (2011). Perceptions of breast health awareness in Black British women. Eur J Oncol Nurs.

[64] Bansal N, Bhopal R, Steiner M, Brewster DH (2012). Major ethnic group differences in breast cancer screening uptake in Scotland are not extinguished by adjustment for indices of geographical residence, area deprivation, long-term illness and education. Br J Cancer.

[65] Forbes L, Atkins L, Thurnham A, Layburn J, Haste F, Ramirez A (2011). Breast cancer awareness and barriers to symptomatic presentation among women from different ethnic groups in East London. Br J Cancer.

[66] Jain A, Acik-Toprak N, Serevitch J, Nazroo J: Inequalities in breast screening uptake among South Asian women in the UK: The role of service providers. *2The Nightingale Centre and Genesis Prevention Centre, University Hospital of South Manchester, Manchester, UK 3Manchester Academic Health Sciences Centre, Institute of Cancer Sciences, Manchester, UK* 2012.

[67] Karbani G, Lim J, Hewison J, Atkin K, Horgan K, Lansdown M, Chu CE (2011). Culture, attitude and knowledge about breast cancer and preventive measures: a qualitative study of South Asian breast cancer patients in the UK. Asian Pac J Cancer Prev.

[68] Renshaw C, Jack RH, Dixon S, Møller H, Davies EA (2010). Estimating attendance for breast cancer screening in ethnic groups in London. BMC Public Health.

[69] Woof VG, Ruane H, Ulph F, French DP, Qureshi N, Khan N, Evans DG, Donnelly LS (2020). Engagement barriers and service inequities in the NHS Breast Screening Programme: Views from British-Pakistani women. J Med Screen.

[70] Ekechi C, Olaitan A, Ellis R, Koris J, Amajuoyi A, Marlow LA (2014). Knowledge of cervical cancer and attendance at cervical cancer screening: a survey of Black women in London. BMC Public Health.

[71] Marlow LA, Waller J, Wardle J (2015). Barriers to cervical cancer screening among ethnic minority women: a qualitative study. Journal of Family Planning and Reproductive Health Care.

[72] Marlow LA, Wardle J, Waller J (2015). Understanding cervical screening non-attendance among ethnic minority women in England. Br J Cancer.

[73] Constantinou P, Dray-Spira R, Menvielle G (2016). Cervical and breast cancer screening participation for women with chronic conditions in France: results from a national health survey. BMC Cancer.

[74] Duffy SW, Tabár L (2021). Yen AM-F, Dean PB, Smith RA, Jonsson H, Törnberg S, Chiu SY-H, Chen SL-S, Jen GH-H: Beneficial effect of consecutive screening mammography examinations on mortality from breast cancer: a prospective study. Radiology.

[75] Dunn SF, Lofters AK, Ginsburg OM, Meaney CA, Ahmad F, Moravac MC, Nguyen CTJ, Arisz AM (2017). Cervical and breast cancer screening after CARES: a community program for immigrant and marginalized women. Am J Prev Med.

[76] Moodley J, Constant D, Mwaka A, Scott S, Walter F (2020). Mapping awareness of breast and cervical cancer risk factors, symptoms and lay beliefs in Uganda and South Africa. PLoS ONE.

[77] Islam RM, Billah B, Hossain MN, Oldroyd J (2017). Barriers to cervical cancer and breast cancer screening uptake in low-income and middle-income countries: a systematic review. Asian Pacific journal of cancer prevention: APJCP.

[78] Karimi SE, Rafiey H, Sajjadi H, Nejad FN (1867). Identifying the social determinants of breast health behavior: A qualitative content analysis. Asian Pacific journal of cancer prevention: APJCP.

[79] Leonard T, Hughes AE, Pruitt SL (2017). Understanding how low–socioeconomic status households cope with health shocks: an analysis of multisector linked data. Ann Am Acad Pol Soc Sci.

[80] Murillo R: Social inequalities in cancer in Latin America. *Reducing social inequalities in cancer: evidence and priorities for research* 2019.

[81] Park MJ, Park E-C, Choi KS, Jun JK, Lee H-Y (2011). Sociodemographic gradients in breast and cervical cancer screening in Korea: the Korean National Cancer Screening Survey (KNCSS) 2005–2009. BMC Cancer.

[82] Black E, Hyslop F, Richmond R (2019). Barriers and facilitators to uptake of cervical cancer screening among women in Uganda: a systematic review. BMC Womens Health.

[83] Idehen EE, Pietilä A-M, Kangasniemi M (2020). Barriers and facilitators to cervical screening among migrant women of African origin: A qualitative study in Finland. Int J Environ Res Public Health.

[84] Adunlin G, Cyrus JW, Asare M, Sabik LM (2019). Barriers and facilitators to breast and cervical cancer screening among immigrants in the United States. J Immigr Minor Health.

[85] de Cuevas RMA, Saini P, Roberts D, Beaver K, Chandrashekar M, Jain A, Kotas E, Tahir N, Ahmed S, Brown SL (2018). A systematic review of barriers and enablers to South Asian women’s attendance for asymptomatic screening of breast and cervical cancers in emigrant countries. BMJ Open.

[86] Ferdous M, Lee S, Goopy S, Yang H, Rumana N, Abedin T, Turin TC (2018). Barriers to cervical cancer screening faced by immigrant women in Canada: a systematic scoping review. BMC Womens Health.

[87] Boom K, Lopez M, Daheri M, Gowen R, Milbourne A, Toscano P, Carey C, Guerra L, Carvajal J, Marin E (2019). Perspectives on cervical cancer screening and prevention: challenges faced by providers and patients along the Texas-Mexico border. Perspect Public Health.

[88] Chidyaonga-Maseko F, Chirwa ML, Muula AS: Underutilization of cervical cancer prevention services in low and middle income countries: a review of contributing factors. *Pan African medical journal* 2015, 21. 10.11604/pamj.2015.21.231.6350 PMC4607967 26523173

[89] Gele AA, Qureshi SA, Kour P, Kumar B, Diaz E (2017). Barriers and facilitators to cervical cancer screening among Pakistani and Somali immigrant women in Oslo: a qualitative study. Int J Women's Health.

[90] Orji AF, Yamashita T (2021). Racial disparities in routine health checkup and adherence to cancer screening guidelines among women in the United States of America. Cancer Causes Control.

[91] Marques P, Nunes M (2020). Antunes MdL, Heleno B, Dias S: Factors associated with cervical cancer screening participation among migrant women in Europe: a scoping review. International journal for equity in health.

[92] Döbrössy B, Girasek E, Susánszky A, Koncz Z, Győrffy Z, Bognár VK (2020). " Clicks, likes, shares and comments" a systematic review of breast cancer screening discourse in social media. PLoS ONE.

[93] Milner GE, McNally RJ (2020). Nonadherence to breast and cervical cancer screening among sexual minority women: Do stigma-related psychological barriers play a role?. Health Psychol.

[94] White-Means S, Dapremont J, Davis BD, Thompson T (2020). Who can help us on this journey? African American woman with breast cancer: Living in a city with extreme health disparities. Int J Environ Res Public Health.

[95] Allen JD, Caspi C, Yang M, Leyva B, Stoddard AM, Tamers S, Tucker-Seeley RD, Sorensen GC (2014). Pathways between acculturation and health behaviors among residents of low-income housing: The mediating role of social and contextual factors. Soc Sci Med.

[96] Jones ME, Schoemaker MJ, Wright LB, Ashworth A, Swerdlow AJ (2017). Smoking and risk of breast cancer in the Generations Study cohort. Breast Cancer Res.

[97] Modibbo FI, Dareng E, Bamisaye P, Jedy-Agba E, Adewole A, Oyeneyin L, Olaniyan O, Adebamowo C (2016). Qualitative study of barriers to cervical cancer screening among Nigerian women. BMJ Open.

[98] Kirubarajan A, Leung S, Li X, Yau M, Sobel M (2021). Barriers and facilitators for cervical cancer screening among adolescents and young people: a systematic review. BMC Womens Health.

[99] Mafiana JJ, Dhital S, Halabia M, Wang X (2022). Barriers to uptake of cervical cancer screening among women in Nigeria: a systematic review. Afr Health Sci.

[100] Katito G, Davies E: Exploring the social-ecological factors related to physical activity participation among Black, Asian and minority ethnic immigrants. *Health Education* 2021.

[101] Akinlotan M, Bolin JN, Helduser J, Ojinnaka C, Lichorad A, McClellan D (2017). Cervical cancer screening barriers and risk factor knowledge among uninsured women. J Community Health.

[102] Getachew S, Getachew E, Gizaw M, Ayele W, Addissie A, Kantelhardt EJ (2019). Cervical cancer screening knowledge and barriers among women in Addis Ababa. Ethiopia PloS one.

[103] Sultana R, Hafeez M, Shafiq S (2019). Awareness about cervical cancer in Pakistani women. PAFMJ.

[104] Alam Z, Shafiee Hanjani L, Dean J, Janda M (2021). Cervical cancer screening among immigrant women residing in Australia: a systematic review. Asia Pacific Journal of Public Health.

[105] Patel H, Sherman SM, Tincello D, Moss EL (2020). Awareness of and attitudes towards cervical cancer prevention among migrant Eastern European women in England. J Med Screen.

[106] Hobbs GS, Landrum MB, Arora NK, Ganz PA, Van Ryn M, Weeks JC, Mack JW, Keating NL (2015). The role of families in decisions regarding cancer treatments. Cancer.

[107] Molina Y, Ornelas IJ, Doty SL, Bishop S, Beresford SA, Coronado GD (2015). Family/friend recommendations and mammography intentions: the roles of perceived mammography norms and support. Health Educ Res.

[108] Adegboyega A, Aleshire M, Dignan M, Hatcher J (2019). Spousal support and knowledge related to cervical cancer screening: Are Sub-Saharan African immigrant men interested?. Health Care Women Int.

[109] Nyblade L, Stockton M, Travasso S, Krishnan S (2017). A qualitative exploration of cervical and breast cancer stigma in Karnataka. India BMC women's health.

[110] Ginjupalli R, Mundaden R, Choi Y, Herfel E, Oketch SY, Watt MH, Makhulo B, Bukusi EA, Huchko M (2022). Developing a framework to describe stigma related to cervical cancer and HPV in western Kenya. BMC Womens Health.

[111] Momenimovahed Z, Tiznobaik A, Taheri S, Hassanipour S, Salehiniya H: A review of barriers and facilitators to mammography in Asian women. *ecancermedicalscience* 2020, 14. 10.3332/ecancer.2020.1146 PMC7738271 33343705

[112] Whale K, Wylde V, Beswick A, Rathbone J, Vedhara K, Gooberman-Hill R (2019). Effectiveness and reporting standards of psychological interventions for improving short-term and long-term pain outcomes after total knee replacement: a systematic review. BMJ Open.

