# Supplementary material for: Barriers to breast and cervical cancer screening uptake among Black, Asian, and Minority Ethnic women in the United Kingdom: evidence from a mixed-methods systematic review
Source: BMC Health Serv Res. 2023 Apr 22;23:390. doi: 10.1186/s12913-023-09410-x (PMC10122823; doi:10.1186/s12913-023-09410-x)
Supplement: Supplementary file 1 — Additional file 1. [file 12913_2023_9410_MOESM1_ESM.pdf]

Supplementary information

Appendix 1: Quality assessment using the MMAT format

S/N	First Author (year)	Q1	Q2	Q3	Q4	Q5	Q6	Total score (%)
1	Renshaw (2010)	Yes	Yes	No	Yes	Yes	Yes	90
2	Jain (2012)	Yes	Yes	No	Yes	Yes	Yes	90
3	Woof (2020b)	Yes	Yes	Yes	Yes	Yes	Yes	100
4	Bansal (2012)	Yes	Yes	Yes	Yes	Yes	Yes	100
5	Forbes (2011)	Yes	Yes	Yes	Yes	Yes	Yes	100
6	Karbani (2011)	Yes	Yes	No	Yes	Yes	Yes	90
7	Bamidele (2017)	Yes	Yes	Yes	Yes	Yes	Yes	100
8	Banning (2011)	Yes	Yes	No	No	Yes	Yes	80
9	Marlow (2017)	Yes	Yes	Yes	Yes	Yes	Yes	100
10	Marlow (2015a)	Yes	Yes	Yes	Yes	Yes	Yes	100
11	Ekechi (2014)	Yes	Yes	Yes	Yes	Yes	Yes	100
12	Marlow (2015b)	Yes	Yes	Yes	Yes	Yes	Yes	100
13	Nelson (2021)	Yes	Yes	Yes	Yes	Yes	Yes	100

Q1: Are there clear qualitative or quantitative research questions (or objectives*), or a clear mixed methods question (or objective*)?

Q2: Do the collected data allow address the research question (objective)? E.g., consider whether the follow-up period is long enough for the outcome to occur (for longitudinal studies or study components).

Q3: Is the sampling strategy relevant to address the quantitative research question?

Q4: Is the sample representative of the population understudy?

Q5: Are measurements appropriate (clear origin, validity known, or standard instrument)?

Q6: Are there complete outcome data (80% or above), an acceptable response rate (60% or above), or an acceptable follow-up?.

Appendix II: Details of the excluded studies and reasons for exclusion

Numbers	First Author (year)	Reason for exclusion	Flow diagram denoted
1	Massat (2015)	The study outcome did not meet the eligibility criteria because the study did not mention any barriers to either breast or cervical cancer screenings	Not on screenings
2	Baird (2021)	This study was a review of findings on barriers and facilitators of breast cancer screening, but a previously published review study was not stated as an inclusion criteria in the current study	Review studies
3	Conolly (2020)	The study focused on barriers and facilitators to cervical cancer screening uptake among men who changed their gender to women	Not on BAME women
4	Vrinten (2016)	The study did not report barriers to breast or cervical screening	Not on barriers
5	Gorman (2016)	The study failed to report the barriers to non-uptake of breast screening in Scotland by the polish women migrants	Not on barriers
6	Jack (2014)	The study only reported the frequency and prevalence of breast cancer screening uptake but did not report the barriers to the uptake	Not on barriers

7	Davies (2013)	No information on barriers to breast cancer screening was reported in the study	Not on screenings
8	Price (2010)	The study compared the frequency of bowel cancer screening with breast cancer screening and no information on barriers to breast cancer screening was reported	Not on barriers
9	Brennan (2017)	This study reported the incidence of breast cancer occurrence in the United Kingdom and Australia but no information on barriers to breast cancer screening uptake was provided	Not on barriers
10	Gathani (2021)	This study provided an update on how breast cancer incidence is disaggregated by ethnicity in the United Kingdom	Not on screenings
11	Woof (2020a)	This is a review study on British-Pakistani women, but a previously published review study was not stated as an inclusion criteria in the current study	Review studies
12	Edgar (2013)	The study was a review study and did not cover BAME women only, and previously published review study was not stated as an inclusion criteria in the current study	Review studies

Appendix III: Search strategies

**Databases**	**Search strategies**	**Results**
**MEDLINE**	((((((("barrier"[All Fields] OR "barrier s"[All Fields] OR "barriers"[All Fields] OR ("factor"[All Fields] OR "factor s"[All Fields] OR "factors"[All Fields]) OR ("associate"[All Fields] OR "associated"[All Fields] OR "associates"[All Fields] OR "associating"[All Fields] OR "association"[MeSH Terms] OR "association"[All Fields] OR "associations"[All Fields]) OR ("analysis"[MeSH Subheading] OR "analysis"[All Fields] OR "determination"[All Fields] OR "determinant"[All Fields] OR "determinants"[All Fields] OR "determinate"[All Fields] OR "determinated"[All Fields] OR "determinates"[All Fields] OR "determinating"[All Fields] OR "determinations"[All Fields] OR "determine"[All Fields] OR "determined"[All Fields] OR "determines"[All Fields] OR "determining"[All Fields])) AND (("breast neoplasms"[MeSH Terms] OR ("breast"[All Fields] AND "neoplasms"[All Fields]) OR "breast neoplasms"[All Fields] OR ("breast"[All Fields] AND "cancer"[All Fields]) OR "breast cancer"[All Fields]) AND ("diagnosis"[MeSH Subheading] OR "diagnosis"[All Fields] OR "screening"[All Fields] OR "mass screening"[MeSH Terms] OR ("mass"[All Fields] AND "screening"[All Fields]) OR "mass screening"[All Fields] OR "early detection of	47
**CINAHL**		38
**BNI**		**16**

<table>
<tr><td>WOS</td><td rowspan="3">cancer"[MeSH Terms] OR ("early"[All Fields] AND "detection"[All Fields] AND "cancer"[All Fields]) OR "early detection of cancer"[All Fields] OR "screen"[All Fields] OR "screenings"[All Fields] OR "screened"[All Fields] OR "screens"[All Fields]))) OR (("uterine cervical neoplasms"[MeSH Terms] OR ("uterine"[All Fields] AND "cervical"[All Fields] AND "neoplasms"[All Fields]) OR "uterine cervical neoplasms"[All Fields] OR ("cervical"[All Fields] AND "cancer"[All Fields]) OR "cervical cancer"[All Fields]) AND ("diagnosis"[MeSH Subheading] OR "diagnosis"[All Fields] OR "screening"[All Fields] OR "mass screening"[MeSH Terms] OR ("mass"[All Fields] AND "screening"[All Fields]) OR "mass screening"[All Fields] OR "early detection of cancer"[MeSH Terms] OR ("early"[All Fields] AND "detection"[All Fields] AND "cancer"[All Fields]) OR "early detection of cancer"[All Fields] OR "screen"[All Fields] OR "screenings"[All Fields] OR "screened"[All Fields] OR "screens"[All Fields]))) AND ("uptake"[All Fields] OR "uptakes"[All Fields] OR "uptaking"[All Fields])) OR ("blackness"[All Fields] OR "blacks"[MeSH Terms] OR "blacks"[All Fields] OR "black"[All Fields]) OR ("asians"[MeSH Terms] OR "asians"[All Fields] OR "asian"[All Fields]) OR ("minority groups"[MeSH Terms] OR ("minority"[All Fields] AND "groups"[All Fields]) OR "minority groups"[All Fields] OR ("minority"[All Fields] AND "group"[All Fields]) OR "minority group"[All Fields])) AND ("united kingdom"[MeSH Terms] OR ("united"[All Fields] AND "kingdom"[All Fields]) OR "united kingdom"[All Fields])) OR ("england"[MeSH Terms] OR "england"[All Fields] OR "england</td><td>46</td></tr>
<tr><td>EMBASE</td><td>36</td></tr>
<tr><td>Scopus</td><td>42</td></tr>
</table>

	s"[All Fields] OR "englands"[All Fields]) OR ("wales"[MeSH Terms] OR "wales"[All Fields] OR "wales s"[All Fields]) OR ("scotland"[MeSH Terms] OR "scotland"[All Fields] OR "scotland s"[All Fields]) OR ("northern ireland"[MeSH Terms] OR ("northern"[All Fields] AND "ireland"[All Fields]) OR "northern ireland"[All Fields])) [from 2010/1/1 to 2022/7/31]	

Appendix IV

Section and Topic	Item #	Checklist item	Location where item is reported
**TITLE**			
Title	1	Identify the report as a systematic review.	Page 1
**ABSTRACT**			
Abstract	2	See the PRISMA 2020 for Abstracts checklist.	Page 1 to 2
**INTRODUCTION**			
Rationale	3	Describe the rationale for the review in the context of existing knowledge.	Page 4 to 5
Objectives	4	Provide an explicit statement of the objective(s) or question(s) the review addresses.	Page 4 to 5
**METHODS**			
Eligibility criteria	5	Specify the inclusion and exclusion criteria for the review and how studies were grouped for the syntheses.	Page 7 to 8
Information sources	6	Specify all databases, registers, websites, organisations, reference lists and other sources searched or consulted to identify studies. Specify the date when each source was last searched or consulted.	Page 5 to 6
Search strategy	7	Present the full search strategies for all databases, registers and websites, including any filters and limits used.	Appendix III
Selection process	8	Specify the methods used to decide whether a study met the inclusion criteria of the review, including how many reviewers screened each record and each report retrieved, whether they worked independently, and if applicable, details of automation tools used in the process.	Page 5 to 9
Data collection process	9	Specify the methods used to collect data from reports, including how many reviewers collected data from each report, whether they worked independently, any processes for obtaining or confirming data from study investigators, and if applicable, details of automation tools used in the process.	Page 5 to 9
Data items	10a	List and define all outcomes for which data were sought. Specify whether all results that were compatible with each outcome domain in each study were sought (e.g. for all measures, time points, analyses), and if not, the methods used to decide which results to collect.	Page 7 to 8
	10b	List and define all other variables for which data were sought (e.g. participant and intervention characteristics, funding sources). Describe any assumptions made about any missing or unclear information.	Page 7 to 8
Study risk of bias assessment	11	Specify the methods used to assess risk of bias in the included studies, including details of the tool(s) used, how many reviewers assessed each study and whether they worked independently, and if applicable, details of automation tools used in the process.	Page 9 to 10
Effect measures	12	Specify for each outcome the effect measure(s) (e.g. risk ratio, mean difference) used in the synthesis or presentation of results.	Page 11
Synthesis methods	13a	Describe the processes used to decide which studies were eligible for each synthesis (e.g. tabulating the study intervention characteristics and comparing against the planned groups for each synthesis (item #5)).	Page 10 to 11
	13b	Describe any methods required to prepare the data for presentation or synthesis, such as handling of missing summary statistics, or data conversions.	Page 10 to 11
	13c	Describe any methods used to tabulate or visually display results of individual studies and syntheses.	Page 10 to

Section and Topic	Item #	Checklist item	Location where item is reported
			11
	13d	Describe any methods used to synthesize results and provide a rationale for the choice(s). If meta-analysis was performed, describe the model(s), method(s) to identify the presence and extent of statistical heterogeneity, and software package(s) used.	Page 10 to 11
	13e	Describe any methods used to explore possible causes of heterogeneity among study results (e.g. subgroup analysis, meta-regression).	Page 10 to 11
	13f	Describe any sensitivity analyses conducted to assess robustness of the synthesized results.	N/A
Reporting bias assessment	14	Describe any methods used to assess risk of bias due to missing results in a synthesis (arising from reporting biases).	N/A
Certainty assessment	15	Describe any methods used to assess certainty (or confidence) in the body of evidence for an outcome.	N/A
**RESULTS**			
Study selection	16a	Describe the results of the search and selection process, from the number of records identified in the search to the number of studies included in the review, ideally using a flow diagram.	Page 12 to 13
	16b	Cite studies that might appear to meet the inclusion criteria, but which were excluded, and explain why they were excluded.	Page 14
Study characteristics	17	Cite each included study and present its characteristics.	Page 13
Risk of bias in studies	18	Present assessments of risk of bias for each included study.	Page 12
Results of individual studies	19	For all outcomes, present, for each study: (a) summary statistics for each group (where appropriate) and (b) an effect estimate and its precision (e.g. confidence/credible interval), ideally using structured tables or plots.	Page 13 to 26
Results of syntheses	20a	For each synthesis, briefly summarise the characteristics and risk of bias among contributing studies.	N/A
	20b	Present results of all statistical syntheses conducted. If meta-analysis was done, present for each the summary estimate and its precision (e.g. confidence/credible interval) and measures of statistical heterogeneity. If comparing groups, describe the direction of the effect.	N/A
	20c	Present results of all investigations of possible causes of heterogeneity among study results.	N/A
	20d	Present results of all sensitivity analyses conducted to assess the robustness of the synthesized results.	N/A
Reporting biases	21	Present assessments of risk of bias due to missing results (arising from reporting biases) for each synthesis assessed.	N/A
Certainty of evidence	22	Present assessments of certainty (or confidence) in the body of evidence for each outcome assessed.	N/A
**DISCUSSION**			
Discussion	23a	Provide a general interpretation of the results in the context of other evidence.	Page 27 to 34
	23b	Discuss any limitations of the evidence included in the review.	Page 35

Section and Topic	Item #	Checklist item	Location where item is reported
	23c	Discuss any limitations of the review processes used.	Page 36
	23d	Discuss implications of the results for practice, policy, and future research.	Page 36
**OTHER INFORMATION**			
Registration and protocol	24a	Provide registration information for the review, including register name and registration number, or state that the review was not registered.	Page 2
	24b	Indicate where the review protocol can be accessed, or state that a protocol was not prepared.	Page 2
	24c	Describe and explain any amendments to information provided at registration or in the protocol.	N/A
Support	25	Describe sources of financial or non-financial support for the review, and the role of the funders or sponsors in the review.	Page 37
Competing interests	26	Declare any competing interests of review authors.	Page 37
Availability of data, code and other materials	27	Report which of the following are publicly available and where they can be found: template data collection forms; data extracted from included studies; data used for all analyses; analytic code; any other materials used in the review.	Page 37

From: Page MJ, McKenzie JE, Bossuyt PM, Boutron I, Hoffmann TC, Mulrow CD, et al. The PRISMA 2020 statement: an updated guideline for reporting systematic reviews. BMJ 2021;372:n71. doi: 10.1136/bmj.n71

For more information, visit: http://www.prisma-statement.org/
